# Development of a self-report questionnaire designed for population-based surveillance of gingivitis in adolescents: assessment of content validity and reliability

**DOI:** 10.1590/1678-7757-2016-0511

**Published:** 2017

**Authors:** Viviana QUIROZ, Daniela REINERO, Patricia HERNÁNDEZ, Johanna CONTRERAS, Rolando VERNAL, Paola CARVAJAL

**Affiliations:** 1 Universidad de Chile, Facultad de Odontología, Departamento de Odontología Conservadora, Santiago, Chile.; 2 Universidad de Chile, Facultad de Odontología, Laboratorio de Biología Periodontal, Santiago, Chile.

**Keywords:** Self report, Surveys and questionnaires, Population surveillance, Periodontal diseases, Gingivitis, Adolescent

## Abstract

The major infectious diseases in Chile encompass the periodontal diseases, with a combined prevalence that rises up to 90% of the population. Thus, the population-based surveillance of periodontal diseases plays a central role for assessing their prevalence and for planning, implementing, and evaluating preventive and control programs. Self-report questionnaires have been proposed for the surveillance of periodontal diseases in adult populations world-wide.

**Objective:**

This study aimed to develop and assess the content validity and reliability of a cognitively adapted self-report questionnaire designed for surveillance of gingivitis in adolescents.

**Material and Methods:**

Ten predetermined self-report questions evaluating early signs and symptoms of gingivitis were preliminary assessed by a panel of clinical experts. Eight questions were selected and cognitively tested in 20 adolescents aged 12 to 18 years from Santiago de Chile. The questionnaire was then conducted and answered by 178 Chilean adolescents. Internal consistency was measured using the Cronbach’s alpha and temporal stability was calculated using the Kappa-index.

**Results:**

A reliable final self-report questionnaire consisting of 5 questions was obtained, with a total Cronbach’s alpha of 0.73 and a Kappa-index ranging from 0.41 to 0.77 between the different questions.

**Conclusions:**

The proposed questionnaire is reliable, with an acceptable internal consistency and a temporal stability from moderate to substantial, and it is promising for estimating the prevalence of gingivitis in adolescents.

## Introduction

Periodontal disease is the most common chronic inflammatory disease detected in humans, affecting nearly 98% of adults over 65 years in Chile^11^. It is a major public health problem due to its high prevalence, its consequences in terms of social, psychological, and economic impacts on individuals, communities, and health services, and its potential prevention and management in terms of alleviation or cure of the disease^2^.

Comprehensive periodontal examination with full-data registration is the gold standard method designed to detect early signs of periodontal disease, allowing us to prevent the destructive forms of the disease; however, its clinical application is expensive and time consuming^9^. Therefore, one must have inexpensive, safe, and easy-to-perform alternative tools to simplify the periodontal data-collection process, which allow the feasibility of the surveillance of the periodontal diseases.

Self-reporting is frequently used as a recording and surveillance method in several pathological conditions and diseases^3,^ ^19^ ^,^ ^20^ ^,^ ^23^ ^,^ ^30^. In adults, self-report questionnaires have been proposed as an epidemiologic tool to analyze the prevalence and incidence of periodontitis^10,^ ^21^ ^,^ ^27^. In fact, a periodontal self-reporting with a set of eight questions has been proposed by the Division of Oral Health at the National Center for Chronic Disease Prevention and Health Promotion and, after its validation, it was applied in different populations^6,8,10,^ ^18^ ^,^ ^24^. With the purpose of evaluating tools that allow the surveillance of periodontal disease at its early stage in younger populations, we analyze the content validity and reliability of a new self-report questionnaire designed to detect gingivitis in adolescents.

## Material and methods

### Experimental design

This cross-sectional study was conducted among Chilean adolescents (12 to 18 years old), who were invited to participate in the study between April and December 2015. The study design (Protocol #2013/26) was approved by the Institutional Ethics Committee and conducted in accordance with the Helsinki Declaration of 1975, as revised in 2000. The protocol of the study was clearly explained to all the participants, who agreed to take part in it by signing an informed consent form reviewed and approved by the institutional committee.

### Preliminary item selection

An initial self-report questionnaire was developed by selecting items related to gingivitis, after reviewing the relevant literature on existing self-reported periodontal measures. Criteria for selecting the self-report items included: having a recognized association with gingival inflammation as a risk indicator or factor; or having face validity as being associated with early signs and symptoms of periodontal disease. A total of 10 items were identified, written in English, and then adapted and translated into Latin-American Spanish, using a backward-forward translational method by two bilingual periodontists (questionnaire version 1).

### Item validation

The validation of the content of each item was performed by a consensus panel of rater experts. This panel consisted of two periodontists, a PhD in public health, a master in pediatric dentistry, and a general dentist expert in adolescents, all of them University Professors and selected considering at least 5 years of clinical expertise, national and international presentations, and research on the referred phenomenon of interest. The analyzed parameters were coherence, relevance, and clarity of each item, as well as whether answer options were appropriate and sufficient. For each parameter, a score of 1 to 9-points was assigned, being 9 very important, 6 important, and 1 unimportant, and when the judges disagreed or an average <5-points was obtained, the item was discarded. After panel consensus and recommendations, questionnaire version 2 was obtained and then pilot tested.

### Questionnaire cognitive evaluation

Pilot test was conducted using 20 adolescent respondents from Santiago de Chile, randomly selected. An answer percentage was calculated and then each answer was discussed considering how the participant understood and processed each question in a focus-group consisting of 10 of these adolescents. A discussion of approximately 60 minutes was conducted, audio-recorded, and transcribed, and data were analyzed by qualitative thematic analysis, in line with the framework approach. This process resulted in 8 cognitively tested questions, which were pooled in a new questionnaire (questionnaire version 3).

### Reliability

The questionnaire version 3 was then applied to the selected adolescents, who voluntarily accepted to participate and autonomously answered the questionnaire in an average time of 10 minutes, in a classroom within the educational establishment. The adolescents were aged between 12 and 18 years old and attended educational establishments in Santiago de Chile. The educational establishments were selected by convenience from different sources of funding (public and private). The sample size was determined following a recommended calculation method^5^ with a power of 90% and confidence level of 95%. Reliability was determined by evaluating the internal consistency of the whole set of self-report items and of each individual question^25^. In addition, temporal stability was also assessed by asking the 30% of the total participants to answer the instrument twice, with a 2-week interval between both applications.

### Data analysis

The inter-judge agreement was determined by their agreement percentage and Kappa-test. The reliability of the questionnaire was estimated by its internal consistency, measured by Cronbach’s alpha, and by its temporal stability, measured by the agreement percentage and by the Kappa-index reached in the test-retest method. Data were analyzed using a statistical software (Stata statistical software v.13, StataCorp, LP, College Station, TX, USA) and a statistical significance was considered when p<0.05.

## Results

### Design of the instrument

**Figure 1:**
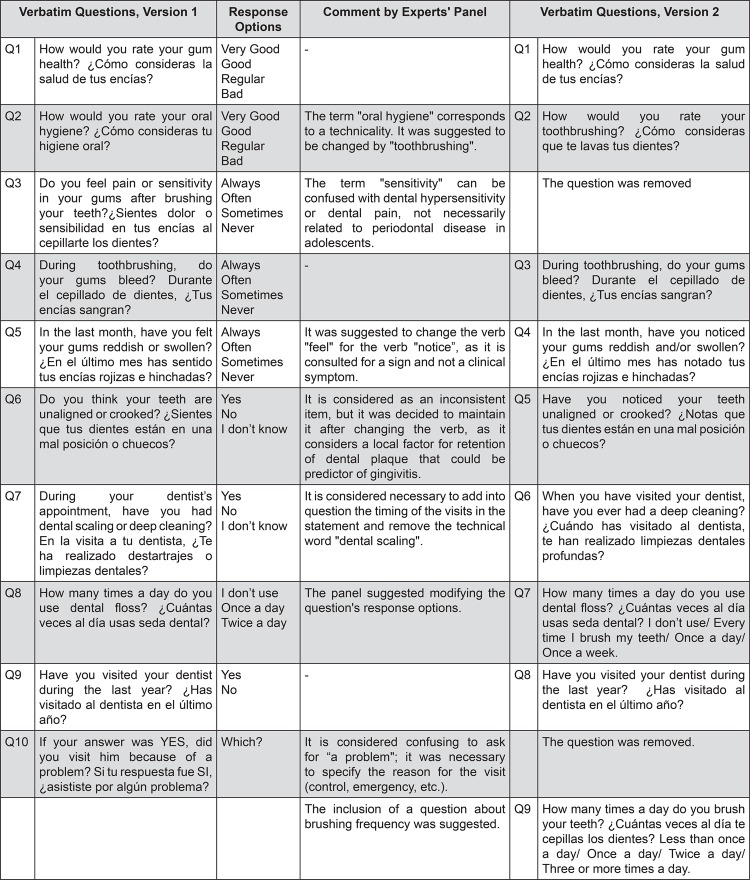
Questionnaire version 1 and version 2 in English and Spanish

Using as a reference the proposed Eke and Dye questionnaire for their cognitive validity in Spanish, the first version of the questionnaire was made (questionnaire version 1)^8,^ ^18^. It was composed of one dimension with 10 self-reports of early on-set of periodontal disease (gingivitis) items, focused on adolescents instead of adults (Figure 1).

### Content validity

The questionnaire version 1 was then evaluated by an expert panel that agreed (>90%) in rating the 10 items as important or critical in the parameters of adequacy, clarity, consistency, and relevance. Following the experts’ written suggestions, items were revised, 2 questions were excluded, and one question was added, obtaining questionnaire version 2 (Figure 1).

### Cognitive evaluation of the instrument

Twenty adolescents answered questionnaire version 2 and each question had a response rate >95%. Content analysis of focus group was made and the cognitively adapted questions were included in questionnaire version 3.

*Q1; How would you rate your gum health?, Q2; How would you rate your toothbrushing?,* and *Q3; During toothbrushing, do your gums bleed?* No problems were identified with these questions. The adolescents said that “diseased gums” are those that bleed during toothbrushing, smell bad, and are red. They associate a good brushing with brushing all the tooth surfaces, leaving them with a smooth texture: “to pass the tongue over them and feel them soft.” Therefore, these items remained in questionnaire version 3.

*Q4; In the last month, have you noticed your gums reddish and/or swollen?* and *Q5; Have you noticed your teeth unaligned or crooked?.* The differences in the way respondents answered depended, mostly, on whether they had seen a dentist in the recent past. One of the participants told he did not know “which is the normal color” for gums and, for another participant, a reddish gum is a gum that bleeds. They reported that Q5 is a subjective question, whose answer is determined by personal expectations; some used the comparison with their peers, a dentist’s opinion, and/or self-examination to answer. The group of researchers, however, decided to keep both items.

*Q6; When you have visited your dentist, have you ever had a deep cleaning?* The term “cleaning” seemed confusing; they associate it with caries removal, whitening, or any other activity performed by a dentist and not necessarily related to periodontal treatment. Therefore, the group decided to remove this question for version 3 of the instrument.

*Q7; How many times a day do you use dental floss?, Q8; Have you visited the dentist in the last year?,* and *Q9; How many times a day do you brush your teeth?* No problems were identified with these questions, thus, they remained in questionnaire version 3.

Table 1 shows questionnaire version 3, detailing the frequency response of all options in each item.

**Table 1:** Questionnaire version 3

Question		Response	Individuals	
		Options	n	%
Q1	How would you rate your gum health? ¿Cómo consideras la salud de tus encías?	Very good	6	3.3
		Good	87	48.8
		Regular	81	45.5
		Bad	4	2.2
Q2	How would you rate your toothbrushing? ¿Cómo consideras que te lavas tus dientes?	Very good	25	14
		Good	105	58.9
		Regular	47	26.4
		Bad	1	0.5
Q3	During toothbrushing, do your gums bleed? Durante el cepillado de dientes, ¿Tus encías sangran?	Always	8	4.4
		Often	15	8.4
		Sometimes	99	55.6
		Never	56	31.4
Q4	In the last month, have you noticed your gums reddish and/or swollen¿ En el último mes has notado tus encías rojizas e hinchadas?	Always	4	2.2
		Often	10	5.6
		Sometimes	57	32
		Never	107	60.1
Q5	Have you noticed your teeth unaligned or crooked? ¿Notas que tus dientes están en una mal posición o chuecos?	Yes	87	48.8
		No	55	30.8
		I don’t know	36	20.2
Q6	How many times a day do you brush your teeth? ¿Cuántas veces al día te cepillas los dientes?	Less than once a day	6	3.3
		Once a day	28	15.7
		Twice a day	97	54.4
		Three or more times a day	47	26.4
Q7	How many times a day do you use dental floss? ¿Cuántas veces al día usas seda dental?	I don’t use	125	70.2
		Every time I brush my teeth	11	6.1
		Once a day	21	11.7
		Once a week	21	11.7
Q8	Have you visited your dentist in the last year? ¿Has visitado al dentista en el último año?	No	77	43.2
		Yes	101	56.7

### Reliability

**Figure 2:**
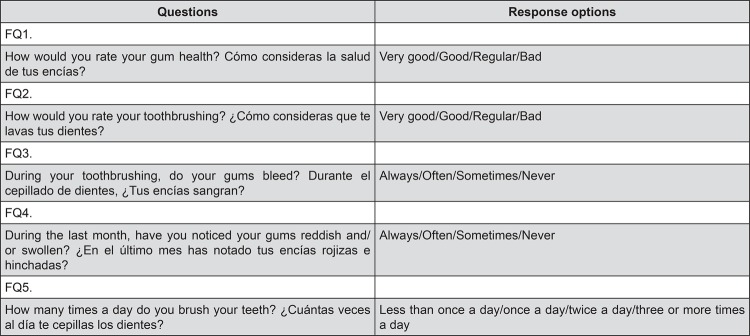
Final version of questionnaire in English and Spanish

Table 2 summarizes the variables and the number of individuals by categories. The median age was 16 years, 66.8% were women, and 75.9%, from a public-funded educational institution. In relation to their habits, 41% reported never having smoked and only 23% reported flossing. All participants completed the questionnaire (Table 1), suggesting that the items were readable and comprehensible, thus providing support to face validity. The percentage of missing responses varied from 0.0% to 0.5% across the items. As seen in Table 3, the reliability measured by its internal consistency (Cronbach’s alpha) increased from 0.64 in questionnaire version 3 to 0.73 in the final version. Average inter-item correlation ranged between 0.44 (Q4) and 0.58 (Q1) for the final version of the questionnaire. Test-retest reliability, measured by Kappa-index, ranged from 0.41 to 0.77 for self-report items. Thus, we obtained the final questionnaire with 5 questions, as shown in Figure 2.

**Table 2:** Study population

	Individuals n=178	
	n	%
Age (Med[min/max])	16 [12/18]	
Sex		
Male	59	33.2
Female	119	66.8
School		
Private	43	24.1
Public	135	75.9
Do you smoke?		
I smoked	71	39.9
I have never smoked	73	41.0
I smoke	34	19.1
Which of the following do you use to clean your teeth?		
Toothbrush	177	99.4
Toothpaste	178	100
Dental Floss	41	23.0
Mouthwash	59	33.1

**Table 3:** Data analysis. *The full questions are shown in Table 1. **p<0.05

Questions*	Questionnaire Version 3		Questionnaire Final Version		Test-retest of Questionnaire Final Version	
	Average inter-item correlation	Cronbach’s α	Average inter-item correlation	Cronbach’s α	Kappa-index**	Agreement (%)
Q1	0.55	0.55	0.58	0.76	0.61	78.1
Q2	0.44	0.58	0.44	0.66	0.46	72.7
Q3	0.45	0.58	0.53	0.72	0.70	83.6
Q4	0.37	0.60	0.44	0.65	0.41	67.2
Q5	0.05	0.68	-	-	-	-
Q6	0.52	0.56	0.47	0.68	0.77	87.2
Q7	0.08	0.67	-	-	-	-
Q8	0.27	0.62	-	-	-	-
Total		0.64		0.73		

## Discussion

In this study, a set of self-reported questionnaire items has been tested for its validity of content and reliability. The final questionnaire had five questions able to be used to do a screening of gingivitis in an adolescent population in Chile. In comparison, the proposed questionnaires to predict the prevalence of periodontitis in adults have a greater number of questions due to the larger amount of signs or symptoms that can be assessed and risk factors that are mostly associated with age, such as the presence of diabetes^6,8,^ ^24^.

The first proposed instrument was subjected to the evaluation of its content validity by a panel of experts. After this instance, the questionnaire underwent changes, by eliminating the question *“Do you feel pain or sensitivity in your gums after brushing your teeth?*,” included within the previously published self-reports^12,13,^ ^27^. This decision came from the recommendation of the expert panel, given that the term “sensitivity” could be confused with dental pain in adolescents and not related to periodontal disease, which is the purpose of the questionnaire. In addition, we noted that the painful symptoms would be mostly associated with the existence of a periodontal pocket or necrotizing forms and therefore, a more advanced stage of the periodontal disease. This could be only slightly prevalent in the target population of this questionnaire. On the other hand, we decided to include the question *“How many times a day do you brush your teeth?*,” despite the fact that its answer could lead to a bias, given its social desirability. In fact, there are reports that have associated less frequent brushing with gingival inflammation and attachment loss^17^. This question remained in the final questionnaire proposed and it showed temporal stability (0.77 Kappa-index). In our sample, 15.7% reported brushing teeth once a day; 54.4%, twice *a* day; and 26.4%, 3 or more times a day, similar to the percentage reported previously^17^.

The value of Cronbach’s alpha was 0.73, showing an acceptable reliability. Other self-report questionnaires for periodontal disease have only reported reliability by temporary stability^7,12,14,15^, unlike our study, which also estimated the internal consistency. In other areas of knowledge that have evaluated behaviors or perceptions about health, however, similar values of reliability have been reported in studies with a larger number of questions and sample size^1,^ ^29^. Both the number of questions in a questionnaire and the sample size determine a higher final value of internal consistency, so the value determined for our questionnaire could even increase if evaluated in a larger sample size. Nevertheless, the internal consistency obtained for this questionnaire by calculating the Cronbach’s alpha is within the expected range, because higher values are not recommended (>0.9), as they may be associated with redundancy between questions, too long instruments, or insufficient representation of the construct to be measured in the instrument^22^.

On the other hand, the temporal stability of the items varied from moderate to substantial^16^, being similar or superior to those reported for other self-report questionnaires^7,12,14^. However, the time between the test and retest in self-reports for periodontal health has ranged from 14 days^15^ to two years^12,14^, making it difficult to compare. In addition, the way to address some of the themes differs: some authors propose only dichotomous responses to questions of presence or absence of an objective clinical sign^7,14,15^. In comparison, this study includes questions focused on perceptions about the health-disease process and its related oral hygiene habits, and has more than two response options for each item, involving a more complex mental process. Also, the variation in the temporal stability of the answers could be attributable to increased awareness and perception of signs and symptoms of disease and habits, leading to a shift towards choosing healthier answers in the retest. This could justify its use as a tool for evaluating preventive programs or oral health education, because this cognitive change could generate a different behavior and subsequent clinical improvement^26^.

Although the question “*In the last month, have you noticed your gums red and/or swollen?*” showed the lowest Kappa value (0.4), an agreement 72% was obtained. This could be explained because only the few individuals who had severe gingivitis safely responded the same answer in both applications of the questionnaire. This disagreement between the Kappa-index and the percentage of agreement could be the result of a characteristic of the statistic used, which is not appropriate for low prevalence conditions, where stability is attributed to chance and not to an existing real stability between applications^28^.

Projections of this study are that this instrument can be validated for an adolescent population and thus promptly detect the disease, as those generated to predict periodontitis in adults. Currently, there are self-report questionnaires that have been validated, but they are designed for an adult population (>18 years) and for signs and consequences of periodontitis or more severe stages of the disease, which would not be useful in a young population and for an early detection of the pathology. In adults, it has been observed that the self-report instrument, with demographic and risk factors items, reached values of sensitivity of 54.6%, specificity of 98%, and an area under the curve (AUC) of 0.93, for predicting severe periodontitis in the observed population^8^ and a sensitivity and specificity of 85% and 58%, respectively, plus an AUC of 0.81, for predicting moderate and severe periodontitis^9^. Indeed, the validation studies of self-report questionnaires conducted so far show good predictive validity for prevalence of severe periodontitis. However, the results and their psychometric properties vary in different populations, needing adaptation and validation for different realities^4^.

The self-report questionnaire designed presented an acceptable reliability for this group of Chilean teenagers. Its criterion validity needs to be assessed on a later stage to determine the correlation of the responses to the questionnaire with the clinical examination (gold standard) and objective signs of gingival inflammation, to validate its use to monitor the prevalence and severity of periodontal diseases at an early age, at population level and at lower cost.

## Conclusions

The results of this analysis suggest that the questionnaire here developed is reliable, with an acceptable internal consistency and a temporal stability from moderate to substantial. In future studies, we recommend researchers to assess its criterion validity and include questions about risk indicators for periodontal disease, to obtain a questionnaire that allows to estimate the prevalence of gingivitis and to evaluate preventive campaigns.

## References

[1] Abanto J, Tsakos G, Paiva SM, Goursand D, Raggio DP, Bönecker M (2013). Cross-cultural adaptation and psychometric properties of the Brazilian version of the scale of oral health outcomes for 5-year-old children (SOHO-5). Health Qual Life Outcomes.

[2] Batchelor P (2014). Is periodontal disease a public health problem?. Br Dent J.

[3] Bergmann MM, Jacobs EJ, Hoffmann K, Boeing H (2004). Agreement of self-reported medical history: comparison of an in-person interview with a self-administered questionnaire. Eur J Epidemiol.

[4] Blicher B, Joshipura K, Eke P (2005). Validation of self-reported periodontal disease: a systematic review. J Dent Res.

[5] Bonett DG (2002). Sample size requirements for testing and estimating coefficient alpha. J Educ Behav Stat.

[6] Cyrino RM, Miranda Cota LO, Pereira Lages EJ, Bastos Lages EM, Costa FO (2011). Evaluation of self-reported measures for prediction of periodontitis in a sample of Brazilians. J Periodontol.

[7] Dolan TA, Gilbert GH, Ringelberg ML, Legler DW, Antonson DE, Foerster U (1997). Behavioral risk indicators of attachment loss in adult Floridians. J Clin Periodontol.

[8] Eke PI, Dye B (2009). Assessment of self-report measures for predicting population prevalence of periodontitis. J Periodontol.

[9] Eke PI, Dye BA, Wei L, Slade GD, Thornton-Evans GO, Beck JD (2013). Self-reported measures for surveillance of periodontitis. J Dent Res.

[10] Eke PI, Genco RJ (2007). CDC Periodontal Disease Surveillance Project: background, objectives, and progress report. J Periodontol.

[11] Gamonal J, Mendoza C, Espinoza I, Muñoz A, Urzúa I, Aranda W (2010). Clinical attachment loss in Chilean adult population: First Chilean National Dental Examination Survey. J Periodontol.

[12] Genco RJ, Falkner KL, Grossi S, Dunford R, Trevisan M (2007). Validity of self-reported measures for surveillance of periodontal disease in two western New York population-based studies. J Periodontology.

[13] Gilbert GH, Litaker MS (2007). Validity of self-reported periodontal status in the Florida dental care study. J Periodontol.

[14] Ho AW, Grossi SG, Dunford RG, Genco RJ (1997). Reliability of a self-reported health questionnaire in a periodontal disease study. J Periodontal Res.

[15] Khader Y, Alhabashneh R, Alhersh F (2015). Development and validation of a self-reported periodontal disease measure among Jordanians. Int Dental J.

[16] Koch GG, Landis JR, Freeman JL, Freeman DH, Lehnen RC (1977). A general methodology for the analysis of experiments with repeated measurement of categorical data. Biometrics.

[17] López R, Fernández O, Jara G, Baelum V (2001). Epidemiology of clinical attachment loss in adolescents. J Periodontol.

[18] Miller K, Eke PI, Schoua-Glusberg A (2007). Cognitive evaluation of self-report questions for surveillance of periodontitis. J Periodontol.

[19] Newell SA, Girgis A, Sanson-Fisher RW, Savolainen NJ (1999). The accuracy of self-reported health behaviors and risk factors relating to cancer and cardiovascular disease in the general population: a critical review. Am J Prev Med.

[20] Okura Y, Urban LH, Mahoney DW, Jacobsen SJ, Rodeheffer RJ (2004). Agreement between self-report questionnaires and medical record data was substantial for diabetes, hypertension, myocardial infarction and stroke but not for heart failure. J Clin Epidemiol.

[21] Page RC, Eke PI (2007). Case definitions for use in population-based surveillance of periodontitis. J Periodontol.

[22] Panayides P (2013). Coefficient alpha: interpret with caution. Eur J Psychol.

[23] Pierannunzi C, Hu SS, Balluz L (2013). A systematic review of publications assessing reliability and validity of the Behavioral Risk Factor Surveillance System (BRFSS), 2004-2011. BMC Med Res Methodol.

[24] Slade GD (2007). Interim analysis of validity of periodontitis screening questions in the Australian population. J Periodontol.

[25] Streiner D, Norman G, Cairney J (2003). Health measurement scales: a practical guide to their development and use.

[26] Taani DQ, Alhaija ESJA (2003). Self-assessed bleeding as an indicator of gingival health among 12-14-year-old children. J Oral Rehabil.

[27] Taylor GW, Borgnakke WS (2007). Self-reported periodontal disease: validation in an epidemiological survey. J Periodontol.

[28] Viera AJ, Garrett JM (2005). Understanding interobserver agreement: the kappa statistic. Fam Med.

[29] Wang M, Yi J, Cai L, Hu M, Zhu X, Yao S (2012). Development and psychometric properties of the health-risk behavior inventory for Chinese adolescents. BMC Med Res Methodol.

[30] Wright FV, Law M, Crombie V, Goldsmith CH, Dent P (1994). Development of a self-report functional status index for juvenile rheumatoid arthritis. J Rheumatol.

